# Supplementary material for: Trends in the Epidemiology of Pneumocystis Pneumonia in Immunocompromised Patients without HIV Infection
Source: J Fungi (Basel). 2023 Jul 31;9(8):812. doi: 10.3390/jof9080812 (PMC10455156; doi:10.3390/jof9080812)
Supplement: Supplementary file 1 [file jof-09-00812-s001.zip › jof-2397561-suppl-updated/Table S1_PCP in autoimmune and inflammatory diseases.pdf]

Table S1. Incidence and risk factors of PCP in patients with autoimmune and inflammatory diseases (AIIDs).

Types of AIIDs	Report years	First author [references]	Country	No. of AIID cases	No. of PCP cases	Main risk factors for PCP
RA	2016	Bruce [112]	UK	17	17	Use of TNF-a inhibitors (adalimumab, etanercept, infliximab and certolizumab)
	2010	Louie [113]	USA	788	788	Immunosuppressive therapies and biologic agents (infliximab and etanercept)
	1999	Ward [114]	USA	39	223	Immunosuppressive medications
	2007	Kaur [70]	USA	49	84	Infliximab therapy
	2015	Mori [115]	Japan	346	346	Antirheumatic therapies (infliximab, etanercept, adalimumab, tocilizumab and abatacept)
	2022	Sonomoto [116]	Japan	26	26	Therapy with b/tsDMARDs
	2017	Mimori [117]	Japan	12	12	Use of glucocorticoids and concomitant respiratory diseases
	2017	Hashimoto [118]	Japan	16	16	Underlying lung diseases and use of corticosteroids, methotrexate and biologics (infliximab, golimumab, abatacept and adalimumab)
	2018	Yukawa [119]	Japan	19	19	High doses of prednisolone and methotrexate
	2008	Tokuda [120]	Japan	14	14	Immunosuppressive therapies
	2017	Mecoli [121]	USA	2	21	High doses of corticosteroids, TNF inhibitors and methotrexate
	2021	Hsu [122]	Taiwan	56	242	Use of immunosuppressants (steroids dose and mycophenolate)
	2014	Fillatre [97]	France	8	154	Impaired immunity due to underlying diseases, corticosteroid therapies
	2017	Li [123]	China	3	52	Immunosuppressive therapies (steroids) and biologic agents (cyclophosphamide, mycophenolate mofetil, methotrexate, cyclosporin A, azathioprine, leflunomide, rituximab, infliximab and tacrolimus).
	1996	Yale [4]	USA	3	116	Systemic corticosteroid therapy
SLE	1994	Godeau [124]	France.	6	34	Treatment with cytotoxic agents and corticosteroids
	1999	Ward [114]	USA	94	223	Immunosuppressive medications
	2009	Lertnawapan	Thailand	15	15	High-dose prednisolone therapy

		[125]				
	2019	Wang [126]	China	9	9	Long-term use of glucocorticoids and the immunosuppressants
	2017	Mecoli [121]	USA	4	21	High-dose corticosteroids and cyclophosphamide
	2008	Gupta [127]	NA	121	121	Use of cyclophosphamide and high-dose corticosteroids, hypocomplementemia
	2020	Yeo [128]	Taiwan	58	58	Use of mycophenolate mofetil/mycophenolic acid and high-dose cyclophosphamide or glucocorticoid
	2017	Li [123]	China	16	52	Use of steroids and other immunosuppressive agents including Cyclophosphamide, mycophenolate mofetil, methotrexate, cyclosporin A, azathioprine, leflunomide, rituximab/infliximab and tacrolimus)
	1996	Yale [4]	USA	1	116	Systemic corticosteroid therapy
	2021	Hsu [122]	Taiwan	74	242	Underlying ARDs and use of immunosuppressants (steroids and mycophenolate)
PsA	2007	Kaur [70]	USA	1	84	Infliximab therapy
	2007	Lahiff [129]	Ireland.	1	1	Treatment with etanercept
	2016	Jobanputra [130]	UK	1	1	Use of G-CSF
AS	2007	Kaur [70]	USA	2	84	Infliximab therapy
Scleroderma	1999	Ward [114]	USA	12	223	Immunosuppressive medications
SS	2021	Hsu [122]	Taiwan	20	242	Underlying ARDs and immunosuppressants (steroids and mycophenolate)
Sarcoid	2017	Mecoli [121]	USA	2	21	High-dose corticosteroids and immunosuppressants (cyclophosphamide and methotrexate)
GPA	1999	Ward [114]	USA	31	22 3	Immunosuppressive medications
	2007	Kaur [70]	USA	2	84	Infliximab therapy
	2017	Mecoli [121]	USA	3	21	High-dose corticosteroids and cyclophosphamide
	1994	Godeau [124]	France.	12	34	Use of corticosteroids and cytotoxic agents
	1991	Sen [40]	USA	1	4	Treatment with cyclophosphamide and prednisone
	1996	Yale [4]	USA	2	116	Systemic corticosteroid therapy
GN	2007	Wen [131]	Taiwan	1	1	Immunosuppressive therapies (corticosteroids and cyclophosphamide)

	1996	Yale [4]	USA	2	116	Systemic corticosteroid therapy
	2012	Yang [132]	Taiwan	7	7	High-dose corticosteroids and cyclophosphamide, cyclosporine A, mycophenolate mofetil and azathioprine)
	2014	Fillatre [97]	France	5	154	Impaired immunity due to underlying disease and corticosteroid therapy
Still's disease	2007	Kaur [70]	USA	1	84	Infliximab therapy
Inflammatory myopathy	1999	Ward [114]	USA	26	223	Immunosuppressive medications
Systemic sclerosis	2017	Mecoli [121]	USA	1	21	High-dose corticosteroids, cyclophosphamide, and methotrexate
	2021	Hsu [122]	Taiwan	7	242	Underlying ARDs and concomitant immunosuppressants (steroid, and mycophenolate)
	2014	Fillatre [97]	France	2	154	Immunosuppression due to underlying disease, and corticosteroid therapy
HSP	2015	Hernández Roca [133]	Spain	1	1	Immunosuppression due to underlying disease and immunosuppressive therapy (cyclophosphamide and prednisone)
PAN	1999	Ward [114]	USA	21	223	Immunosuppressive medications
	2017	Mecoli [121]	USA	1	21	High-dose corticosteroids and cyclophosphamide
	1977	Bungo [134]	USA	1	1	Immunosuppressive therapies (high doses of corticosteroids, cyclophosphamide and methotrexate)
	2014	Fillatre [97]	France	3	154	Immunosuppression due to the underlying disease and use of corticosteroids.
	1994	Godeau [124]	France	4	34	Treatment with cytotoxic agents and corticosteroids
	1996	Yale [4]	USA	1	116	Systemic corticosteroid therapy
	1991	Sen [40]	USA	1	4	Treated with cyclophosphamide and prednisone
Myositis	2007	Kaur [70]	USA	2	84	Infliximab therapy
	2021	Sabbagh [135]	USA.	13	13	Immunosuppressive therapy, and existing interstitial lung disease.
	2017	Mecoli [121]	USA	4	21	High-dose corticosteroids and cyclophosphamide
	1994	Godeau [124]	France	5	34	Treatment with cytotoxic agents and corticosteroids
	2021	Hsu [122]	Taiwan	21	242	Underlying ARDs, and immunosuppressants (steroid dose and mycophenolate)
	2014	Fillatre [97]	France	2	154	Immunosuppression due to the underlying disease, and use of corticosteroids
	2017	Li [123]	China	6	52	Use of steroids and other immunosuppressive agents cyclophosphamide,

						mycophenolate mofetil, methotrexate, cyclosporin A, azathioprine, leflunomide, rituximab/infliximab, and tacrolimus)
	1996	Yale [4]	USA	2	116	Systemic corticosteroid therapy
IBD	2014	Fillatre [97]	France	1	154	Immunosuppression due to the underlying disease, and use of corticosteroids
	2007	Kaur [70]	USA	16	84	Infliximab therapy
	2017	Cotter [136]	USA	4	4	Use of corticosteroids, immune-suppressive medications, and biologics
	2017	Lawrence [137]	NA	92	92	Immunosuppressive therapies (corticosteroids, infliximab, thiopurines, anti-TNF agents and calcineurin inhibitors)
	2022	Schwartz [138]	USA	180	180	Underlying congenital immunodeficiency, and use of corticosteroids
	2019	Yoshida [139]	Japan	28	28	Treatment with steroid, thiopurine, calcineurin inhibitor, and anti-TNF agents
	2013	Long [140]	USA	38	38	Corticosteroid therapy
	2022	Nam [141]	Korea	6 (UC)	6	Corticosteroid therapy and other immunosuppressive therapies (infliximab, azathioprine, and adalimumab)
	2010	Lawrance [142]	Australia & New Zealand	2 (CD)	N/A	Immunosuppressive therapies (mycophenolate mofetil, methotrexate and prednisolone) and use of infliximab and adalimumab
Polyneuritis	2014	Fillatre [97]	France	2	154	Immunosuppression due to underlying diseases, and use of corticosteroids
Systemic vasculitis	1996	Yale [4]	USA	1	116	Systemic corticosteroid therapy
Chronic hepatitis	1996	Yale [4]	USA	1	116	Systemic corticosteroid therapy

Abbreviations: TNF, tumor necrosis factor ; b/tsDMARDs, biologic and targeted synthetic disease-modifying antirheumatic drugs; ARDs, autoimmune rheumatic diseases; SLE, systemic lupus erythematosus; RA, rheumatoid arthritis; AS, ankylosing spondylitis; PsA, psoriatic arthritis; SS, Sjogren syndrome; GPA, granulomatosis with polyangiitis (also known as Wegener's granulomatosis); GN, glomerulonephritis; myositis including polymyositis (PM) and dermatomyositis (DM); HSP, Henoch-Schönlein purpura; UC, ulcerative colitis;; PAN, polyarteritis nodosa; PSL, prednisolone; IBD: inflammatory bowel disease, including Crohn's disease (CD) and ulcerative colitis (UC); NA, not available.
