# Supplementary material for: Trends in the Epidemiology of Pneumocystis Pneumonia in Immunocompromised Patients without HIV Infection
Source: J Fungi (Basel). 2023 Jul 31;9(8):812. doi: 10.3390/jof9080812 (PMC10455156; doi:10.3390/jof9080812)
Supplement: Supplementary file 1 [file jof-09-00812-s001.zip › jof-2397561-suppl-updated/Table S3_PCP in COVID-19.pdf]

Table S3. Reports of co-infection with *P. jirovecii* and SARS-CoV-2 in patients with or without HIV/AIDS.

Countries	Total No. of cases	No. of cases		References
		Without HIV/AIDS	With HIV/AIDS	
France	34	34	0	[228-232]
Italy	23	22	1	[224,225,233-237]
Pakistan	10	10	0	[238]
Greece	8	8	0	[239]
India	6	6	0	[15,240]
US	6	3	3	[241-246]
China	4	2	2	[247-249]
Spain	4	2	2	[250-253]
UK	4	1	3	[254-257]
Canada	3	3	0	[226]
Jordan	3	3	0	[258]
Japan	3	2	1	[259,260]
Iran	2	2	0	[261,262]
South Africa	2	0	2	[263,264]
Romania	1	1	0	[265]
Poland	1	1	0	[266]
Morocco	1	1	0	[267]
Germany	1	0	1	[268]
Argentina	1	0	1	[269]
Ireland	1	0	1	[270]
Indonesia	1	0	1	[271]
