# Supplementary material for: Trends in the Epidemiology of Pneumocystis Pneumonia in Immunocompromised Patients without HIV Infection
Source: J Fungi (Basel). 2023 Jul 31;9(8):812. doi: 10.3390/jof9080812 (PMC10455156; doi:10.3390/jof9080812)
Supplement: Supplementary file 1 [file jof-09-00812-s001.zip › jof-2397561-suppl-updated/Table S4_PCP outbreaks in transplant patients.pdf]

Table S4. Reports of PCP outbreaks in transplant patients.

First author [reference]	Year of report	Country	No. of patients	Duration of outbreaks (months)	Transplanted organs	Genotyping methods	No. of *P. jirovecii* strains/clusters
Hardy [310]	1984	USA	14	19	Kidney	NA	NA
Hennequin [311]	1995	France	7	6	Kidney	NA	NA
Olsson [312]	2001	Sweden	10	8	Kidney	mtLSU	3
Hocker [313]	2005	Germany	7	5	Kidney	MLST (ITS1, 26S rRNA, mtLSU and *β-tub*)	5
de Boer [314]	2007	Netherlands	8	11	Kidney	ITS1 and ITS2	>5
Schmoldt [315]	2008	Germany	16	8	Kidney	MLST (ITS1, *β-tub*, 26S rRNA and mtLSU)	1
Yazaki [316]	2009	Japan	27	22	Kidney	ITS1 and ITS2	1
Arichi [317]	2009	Japan	9	7	Kidney	mtLSU	5
Gianella [318]	2010	Switzerland	20	20	Kidney	MLST (ITS1, 26S rRNA, mtLSU and *β-tub*)	1
Wynckel [319]	2011	France	17	12	Kidney	ITS1 and ITS2	2
Phipps [320]	2011	Australia	14	9	Kidney	MLST (ITS, mtLSU, *β-tub* and *dhps*)	NA
Thomas [321]	2011	UK	21*	21	Kidney	MLST (ITS, mtLSUr)	>5
			11*	12	Kidney	MLST (ITS, mtLSU)	>5
Pliquett [322]	2012	Germany	17	60	Kidney	MLST (ITS1, *β-tub*, 26S rRNA and mtLSU)	3
Brunot [323]	2012	France	7	9	Kidney	NA	1
Chapman [324] and Nankivell [325]	2013	Australia	83	24	Kidney	MLST (ITS, mtLSU, *β-tub* and *dhps*)	NA
Rostvet [326]	2013	Denmark	29	48	Kidney and	Msg-RFLP and MLST (ITS1,	3

					liver	ITS2, 26S rRNA and mtLSU)	
Debourgogne [327]	2014	France	13	52	Kidney	MLST(ITS1, mtLSU, 26S rRNA and *β-tub*)	2
Chandola [328]	2014	India	7	4	Kidney	NA	NA
Gits-Muselli [329]	2015	France	6	26	Kidney	VNTR	1
Desoubeaux [330]	2016	France	4	5	Liver	MLST (*sod*, mtLSU and *cob*)	1
Mulpuru [331]	2016	Canada	10	12	Kidney	ITS1 and ITS2	1
Urabe [332]	2016	Japan	8	NA	Kidney	MLST (*β-tub,*mtLSU, *sod* and *cob*)	1
Inkster [333]	2017	UK	24	15	Kidney	MLST (ITS1 and mtLSU)	2
Vindrios [107]	2017	France	7	5	Heart	MLST (mtLSU, *sod* and *cob)*	1
Robin [334]	2017	France	12	7	Stem cell	VNTR	5
Frealle [335]	2017	France	5	33	Kidney	mtLSU	2
Alanio [336]	2017	France	13	NA	Kidney	VNTR	1
		Belgium	5	NA	Kidney	VNTR	1
		UK	2	NA	Kidney	VNTR	1
Wintenberger [337] and Charpentier [338]	2017	France	12	10	Lung, kidney, heart, liver	NGS-MLST (*cob*, mtLSU and *sod*)	9
Nevez [339]	2018	France	22	14	Kidney	MLST (*dhps,* ITS1, ITS2 and mtLSU)	1
Miguel Montanes [77]	2018	France	15	32	Liver	NA	NA
Ricci [340]	2018	Brazil	17	8	Kidney	MLST (ITS1, ITS2, mtLSU, 26S rRNA and *dhps)*	5
Veronese [341]	2018	Italy	6	10	Heart	MLST (*β-tub*, *dhps*, mtLSU, ITS1 and ITS2)	2
Szydlowicz [342]	2019	Poland	8	33	Kidney	MLST (*mtLSU*, cob, *dhps* and *sod*)	>5

Hosseini-Moghaddam [343]	2020	France	10	23	Heart, kidney, liver	MLST (mtLSU, co*b, sod* and *dhps*)	1
Azar [58]	2022	USA	19	11	Kidney	NGS-MLST** and msg-RFLP	5

This table includes all reports of PCP outbreaks in transplant recipients shown in the heatmap in Figure 4. Reports without genotyping results are excluded in Table 3 in the main text to allow better visualization.

Abbreviations: NA, not available; ITS1, internal transcribed spacer 1; ITS2, internal transcribed spacer 2; mtLSU, mitochondrial large subunit rRNA; mtSSU, mitochondrial small subunit ribosomal RNA; Msg-RFLP, major surface glycoprotein (msg) gene-restriction fragment length polymorphism; UCS-VNTR, msg upstream conserved sequence-variable number of tandem repeats. MLST, multilocus sequence typing; β-tub, beta-tubulin; *sod*, superoxide dismutase; *cob*, cytochrome b; *dhps*, dihydropteroate synthase; 26S rRNA, nuclear 26S rRNA gene; NGS, next-generation sequencing.

* Involved in two separate outbreaks in different hospitals.

**Including mtLSU, PNC (mitochondrial polymorphic non-coding region), ITS1-5.8S-ITS2, 26S rRNA, *sod*, *dhps*, full mitogenome and nuclear rRNA operon.
